# Supplementary material for: The nuclear periphery confers repression on H3K9me2-marked genes and transposons to shape cell fate
Source: Nat Cell Biol. 2025 Jul 22;27(8):1311–26. doi: 10.1038/s41556-025-01703-z (PMC12339402; doi:10.1038/s41556-025-01703-z)
Supplement: Supplementary file 1 — Supplementary Figs. 1–8. [file 41556_2025_1703_MOESM1_ESM.pdf]

nature cell biology

Supplementary information https://doi.org/10.1038/s41556-025-01703-z

The nuclear periphery confers repression on H3K9me2-marked genes and transposons to shape cell fate

In the format provided by the authors and unedited

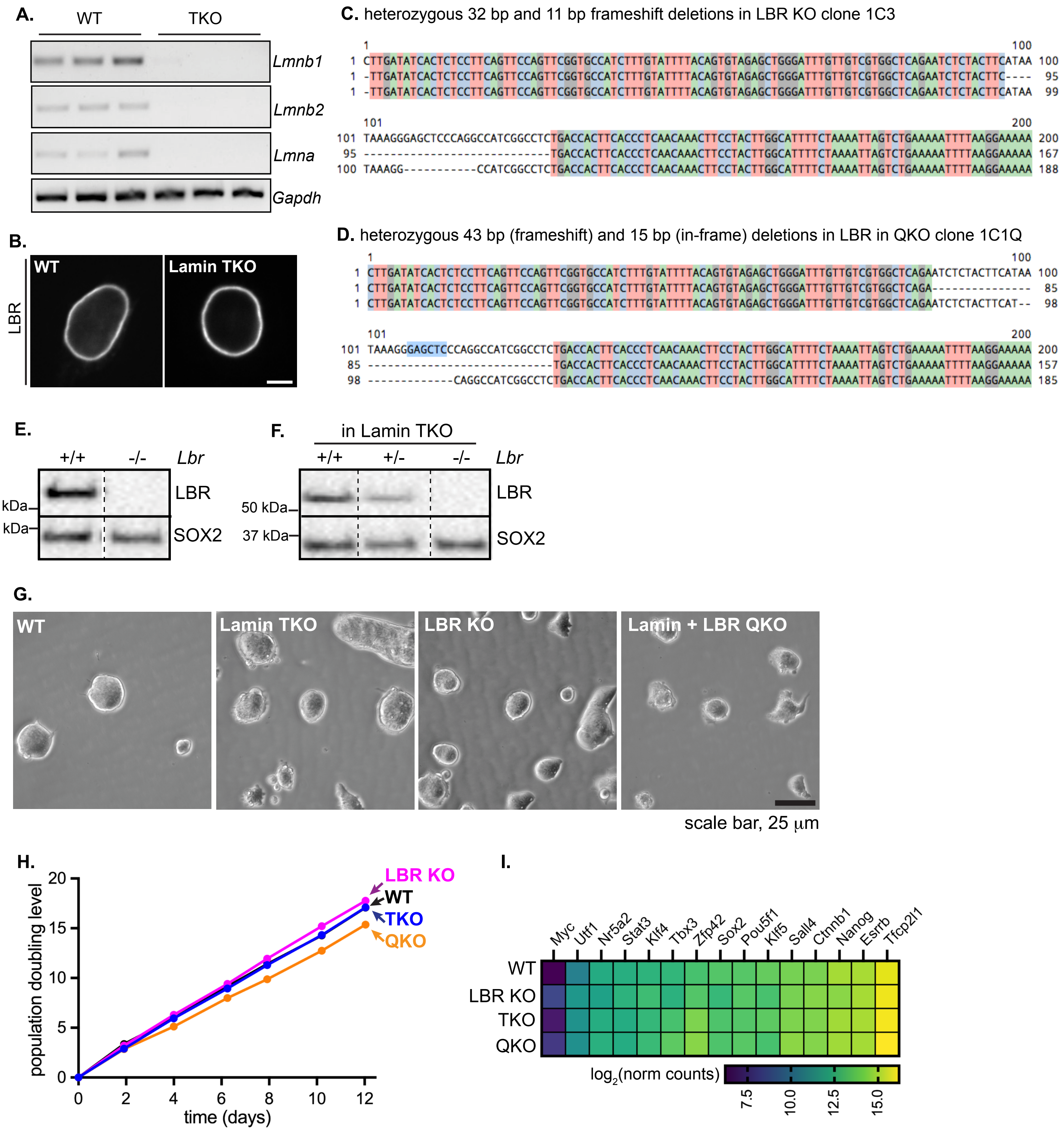
A.
WT
TKO
Lmnb1
Lmnb2
Lmna
Gapdh
B.
WT
Lamin TKO
LBR
C. heterozygous 32 bp and 11 bp frameshift deletions in LBR KO clone 1C3
1 CTTGATATCACTCTCCTTCAGTTCCAGTTCGGTGCCATCTTTGTATTTTACAGTGTAGAGCTGGGATTTGTTGTCGTGGCTCAGAATCTCTACTTCATAA 100
1 -TTGATATCACTCTCCTTCAGTTCCAGTTCGGTGCCATCTTTGTATTTTACAGTGTAGAGCTGGGATTTGTTGTCGTGGCTCAGAATCTCTACTTC---- 95
1 -TTGATATCACTCTCCTTCAGTTCCAGTTCGGTGCCATCTTTGTATTTTACAGTGTAGAGCTGGGATTTGTTGTCGTGGCTCAGAATCTCTACTTCATAA 99
101 TAAAGGGAGCTCCCAGGCCATCGGCCTCTGACCACTTCACCCTCAACAAACTTCCTACTTGGCATTTTCTAAAATTAGTCTGAAAAATTTTAAGGAAAAA 200
95 ----------------------------TGACCACTTCACCCTCAACAAACTTCCTACTTGGCATTTTCTAAAATTAGTCTGAAAAATTTTAAGGAAAAA 167
100 TAAAGG-----------CCATCGGCCTCTGACCACTTCACCCTCAACAAACTTCCTACTTGGCATTTTCTAAAATTAGTCTGAAAAATTTTAAGGAAAAA 188
D. heterozygous 43 bp (frameshift) and 15 bp (in-frame) deletions in LBR in QKO clone 1C1Q
1 CTTGATATCACTCTCCTTCAGTTCCAGTTCGGTGCCATCTTTGTATTTTACAGTGTAGAGCTGGGATTTGTTGTCGTGGCTCAGAATCTCTACTTCATAA 100
1 CTTGATATCACTCTCCTTCAGTTCCAGTTCGGTGCCATCTTTGTATTTTACAGTGTAGAGCTGGGATTTGTTGTCGTGGCTCAGA--------------- 85
1 CTTGATATCACTCTCCTTCAGTTCCAGTTCGGTGCCATCTTTGTATTTTACAGTGTAGAGCTGGGATTTGTTGTCGTGGCTCAGAATCTCTACTTCAT-- 98
101 TAAAGGGAGCTCCCAGGCCATCGGCCTCTGACCACTTCACCCTCAACAAACTTCCTACTTGGCATTTTCTAAAATTAGTCTGAAAAATTTTAAGGAAAAA 200
85 ----------------------------TGACCACTTCACCCTCAACAAACTTCCTACTTGGCATTTTCTAAAATTAGTCTGAAAAATTTTAAGGAAAAA 157
98 -------------CAGGCCATCGGCCTCTGACCACTTCACCCTCAACAAACTTCCTACTTGGCATTTTCTAAAATTAGTCTGAAAAATTTTAAGGAAAAA 185
E.
+/+
-/-
Lbr
LBR
SOX2
50 kDa
37 kDa
F.
in Lamin TKO
+/+
+/-
-/-
Lbr
LBR
SOX2
50 kDa
37 kDa
G.
WT
Lamin TKO
LBR KO
Lamin + LBR QKO
scale bar, 25 µm
H.
population doubling level
time (days)
0
2
4
6
8
10
12
5
15
20
LBR KO
WT
TKO
QKO
I.
Myc
Utf1
Nr5a2
Stat3
Klf4
Tbx3
Zfp42
Sox2
Pou5f1
Klf5
Sall4
Ctnnb1
Nanog
Esrrb
Tfcp2l1
WT
LBR KO
TKO
QKO
log2(norm counts)
7.5
10.0
12.5
15.0

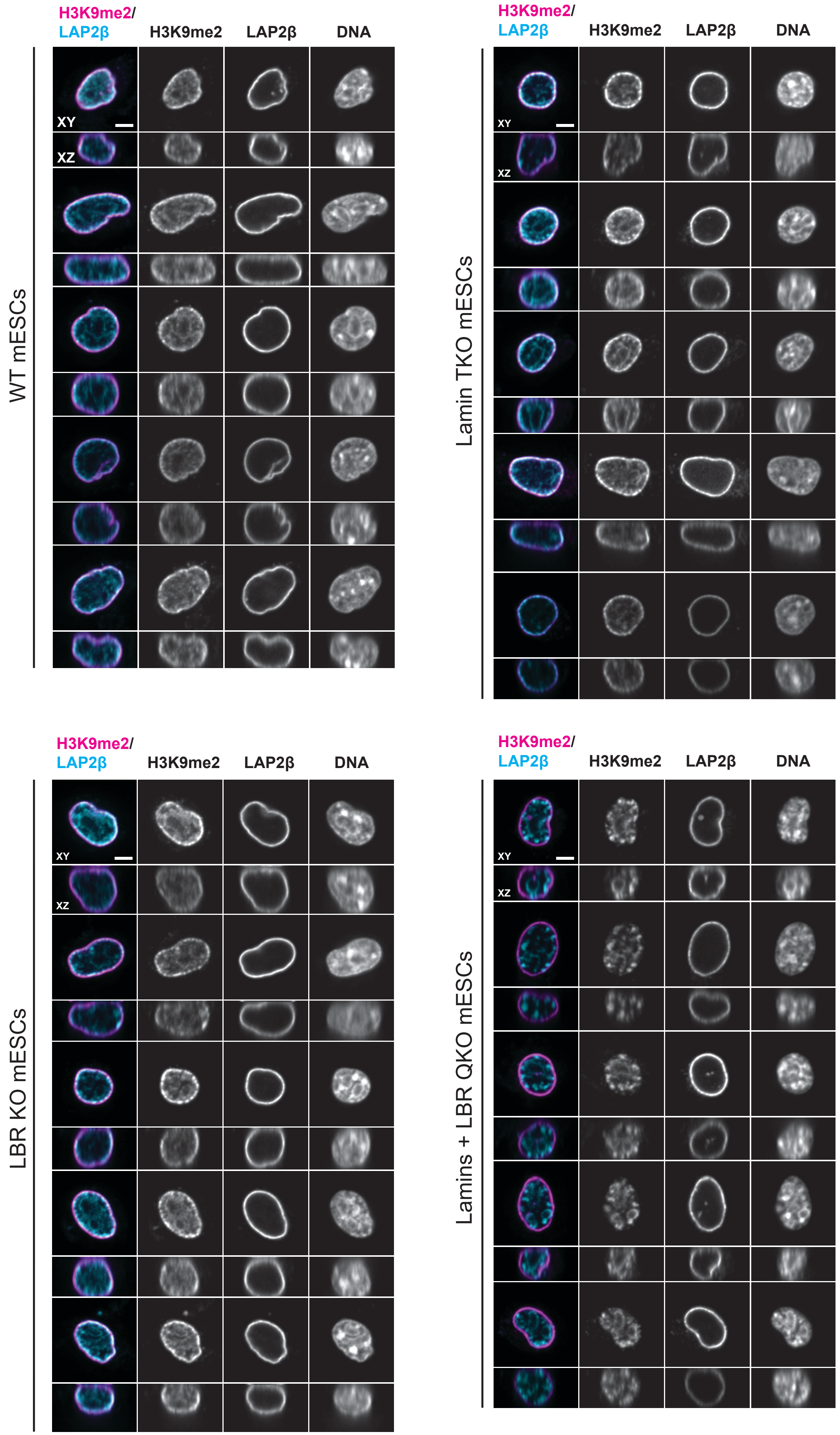

H3K9me2/
LAP2β
H3K9me2
LAP2β
DNA
XY
XZ
WT mESCs
Lamin TKO mESCs
LBR KO mESCs
Lamins + LBR QKO mESCs

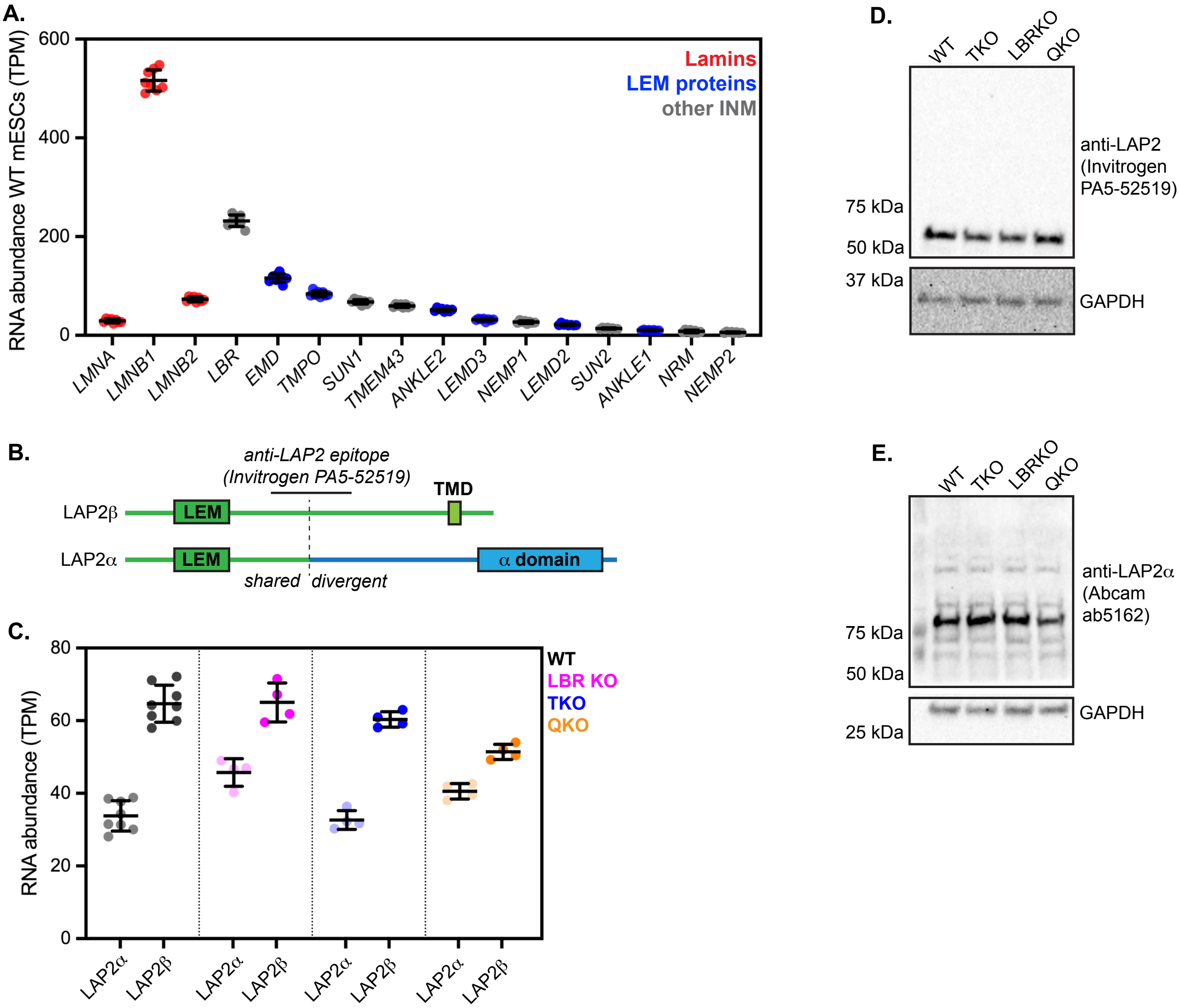
A.
Lamins
LEM proteins
other INM
RNA abundance WT mESCs (TPM)
600
400
200
0
LMNA
LMNB1
LMNB2
LBR
EMD
TMPO
SUN1
TMEM43
ANKLE2
LEMD3
NEMP1
LEMD2
SUN2
ANKLE1
NRM
NEMP2
B.
anti-LAP2 epitope
(Invitrogen PA5-52519)
TMD
LAP2β
LEM
LAP2α
LEM
α domain
shared
divergent
C.
RNA abundance (TPM)
80
60
40
20
0
WT
LBR KO
TKO
QKO
LAP2α
LAP2β
LAP2α
LAP2β
LAP2α
LAP2β
LAP2α
LAP2β
D.
WT
TKO
LBRKO
QKO
anti-LAP2
(Invitrogen
PA5-52519)
75 kDa
50 kDa
37 kDa
GAPDH
E.
WT
TKO
LBRKO
QKO
anti-LAP2α
(Abcam
ab5162)
75 kDa
50 kDa
GAPDH
25 kDa

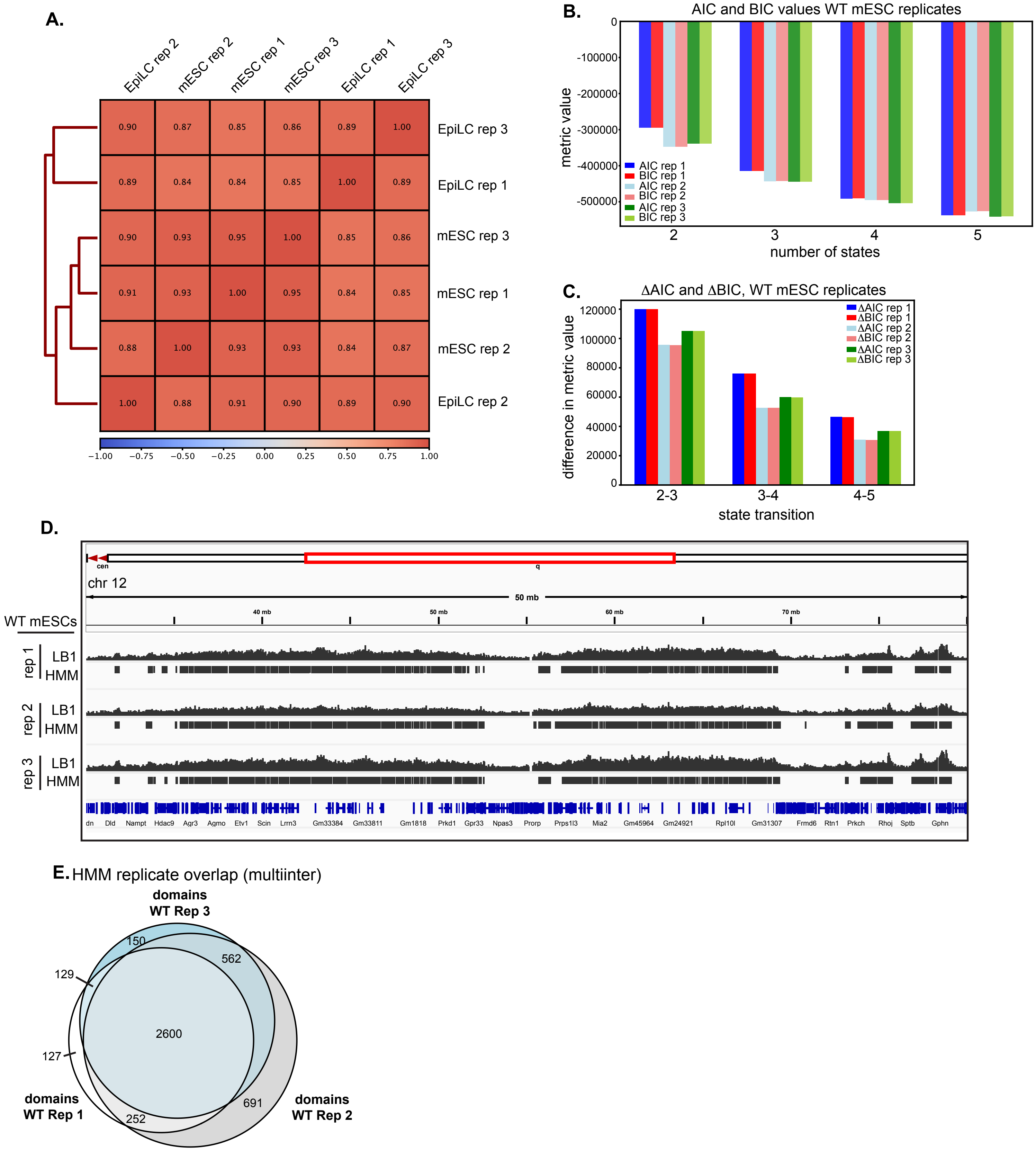
A.
EpiLC rep 2
mESC rep 2
mESC rep 1
mESC rep 3
EpiLC rep 1
EpiLC rep 3
0.90 0.87 0.85 0.86 0.89 1.00 EpiLC rep 3
0.89 0.84 0.84 0.85 1.00 0.89 EpiLC rep 1
0.90 0.93 0.95 1.00 0.85 0.86 mESC rep 3
0.91 0.93 1.00 0.95 0.84 0.85 mESC rep 1
0.88 1.00 0.93 0.93 0.84 0.87 mESC rep 2
1.00 0.88 0.91 0.90 0.89 0.90 EpiLC rep 2
−1.00 −0.75 −0.50 −0.25 0.00 0.25 0.50 0.75 1.00
B.
AIC and BIC values WT mESC replicates
metric value
0
-100000
-200000
-300000
-400000
-500000
AIC rep 1
BIC rep 1
AIC rep 2
BIC rep 2
AIC rep 3
BIC rep 3
2
3
4
5
number of states
C.
ΔAIC and ΔBIC, WT mESC replicates
difference in metric value
120000
100000
80000
60000
40000
20000
0
ΔAIC rep 1
ΔBIC rep 1
ΔAIC rep 2
ΔBIC rep 2
ΔAIC rep 3
ΔBIC rep 3
2-3
3-4
4-5
state transition
D.
cen
q
chr 12
50 mb
40 mb
50 mb
60 mb
70 mb
WT mESCs
rep 1 LB1 HMM
rep 2 LB1 HMM
rep 3 LB1 HMM
dn Dld Nampt Hdac9 Agr3 Agmo Etv1 Scin Lrrn3 Gm33384 Gm33811 Gm1818 Prkd1 Gpr33 Npas3 Prorp Prps1l3 Mia2 Gm45964 Gm24921 Rpl10l Gm31307 Frmd6 Rtn1 Prkch Rhoj Sptb Gphn
E. HMM replicate overlap (multiinter)
domains WT Rep 3
150
562
129
2600
127
691
252
domains WT Rep 1
domains WT Rep 2

A.

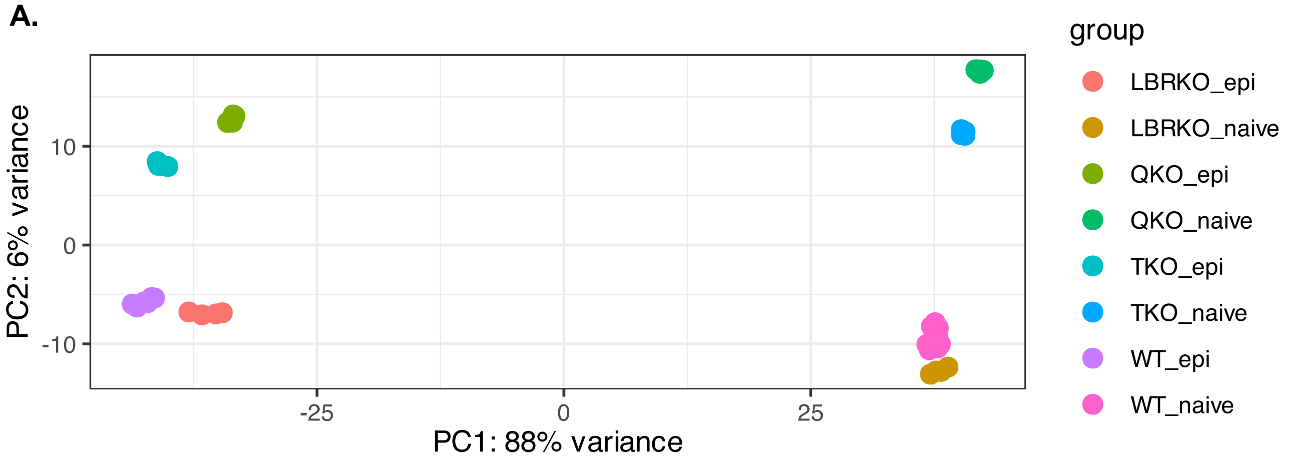

PC2: 6% variance
10
0
-10
-25
0
25
PC1: 88% variance
group
LBRKO_epi
LBRKO_naive
QKO_epi
QKO_naive
TKO_epi
TKO_naive
WT_epi
WT_naive

B.

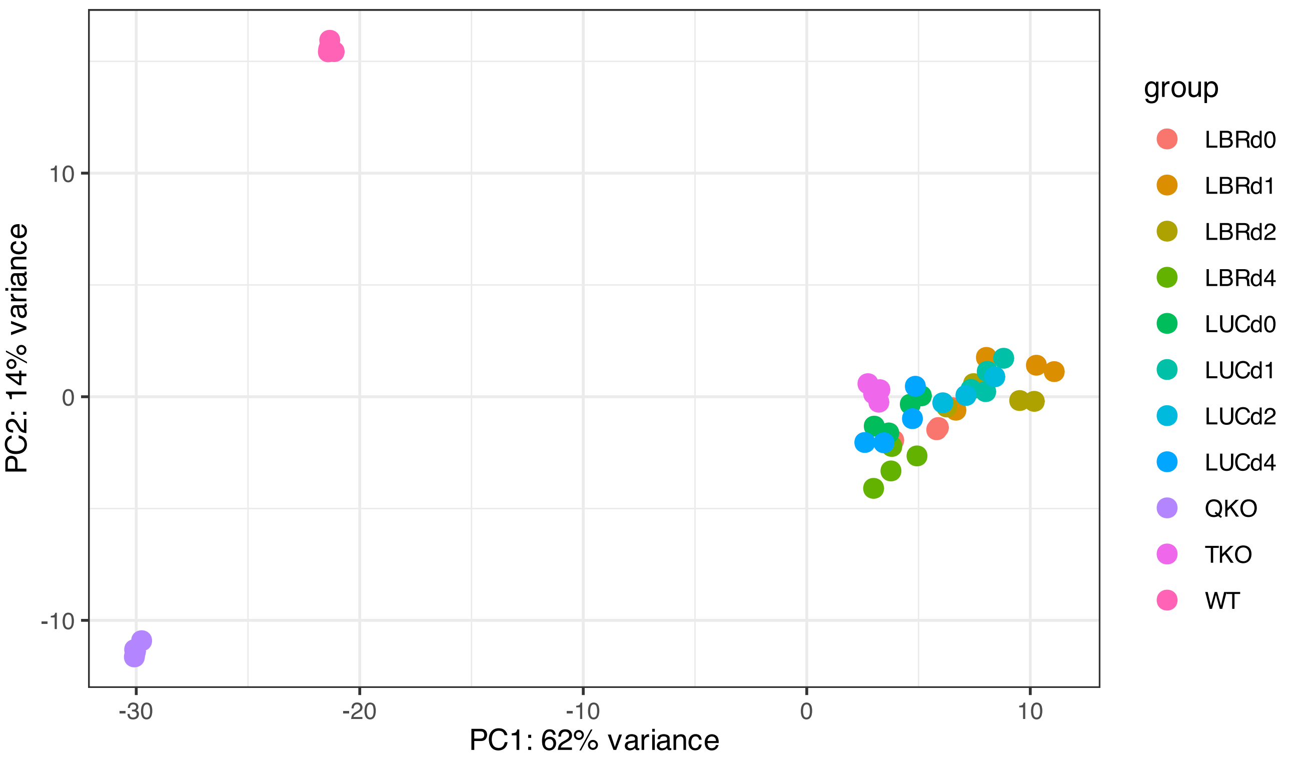

PC2: 14% variance
10
0
-10
-30
-20
-10
0
10
PC1: 62% variance
group
LBRd0
LBRd1
LBRd2
LBRd4
LUCd0
LUCd1
LUCd2
LUCd4
QKO
TKO
WT

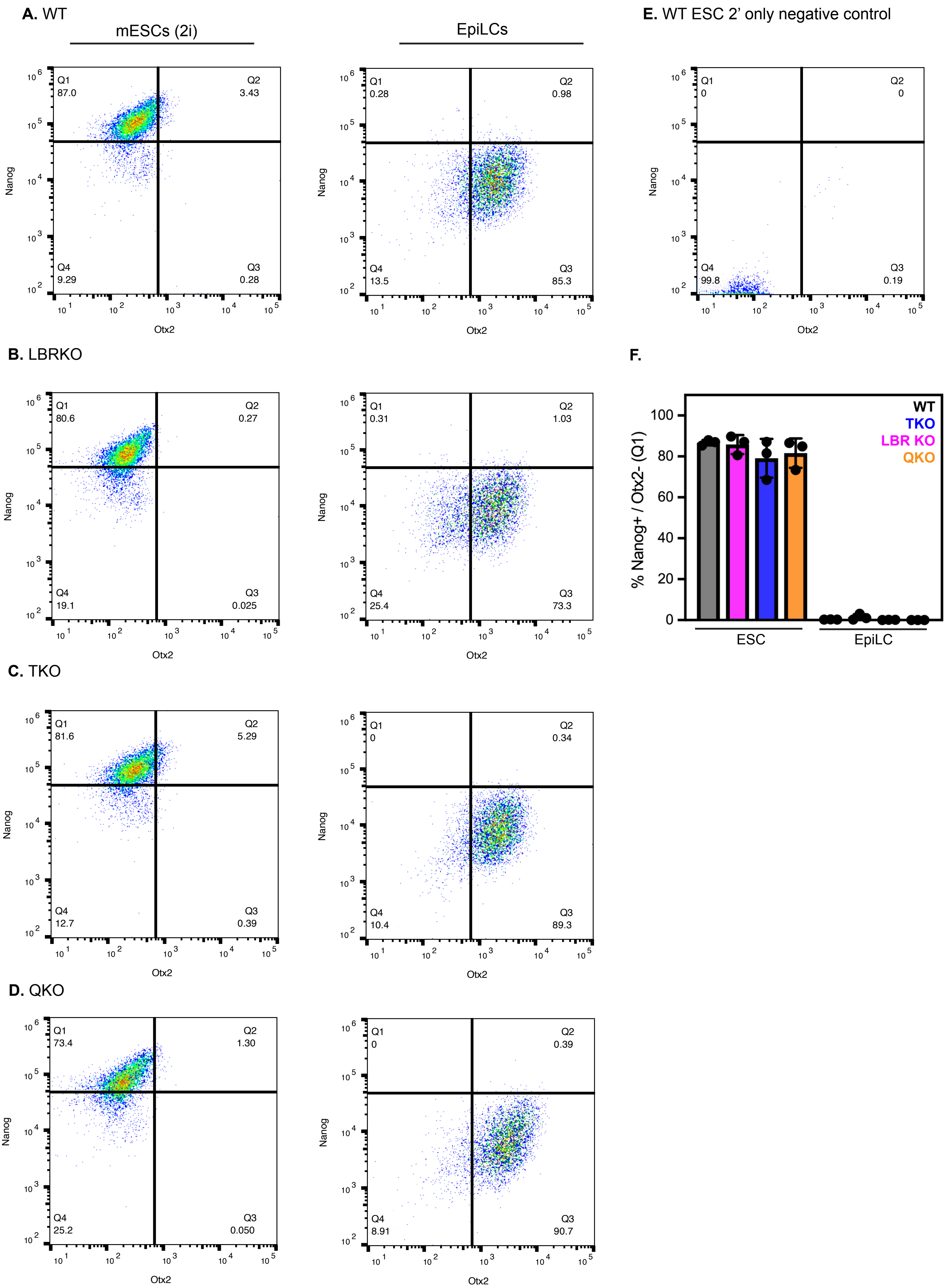
A. WT
mESCs (2i)
EpiLCs
E. WT ESC 2' only negative control
Q1
87.0
Q2
3.43
Q4
9.29
Q3
0.28
Q1
0.28
Q2
0.98
Q4
13.5
Q3
85.3
Q1
0
Q2
0
Q4
99.8
Q3
0.19
Nanog
Otx2
B. LBRKO
Q1
80.6
Q2
0.27
Q4
19.1
Q3
0.025
Q1
0.31
Q2
1.03
Q4
25.4
Q3
73.3
F.
% Nanog+ / Otx2- (Q1)
100
80
60
40
20
0
WT
TKO
LBR KO
QKO
ESC
EpiLC
C. TKO
Q1
81.6
Q2
5.29
Q4
12.7
Q3
0.39
Q1
0
Q2
0.34
Q4
10.4
Q3
89.3
D. QKO
Q1
73.4
Q2
1.30
Q4
25.2
Q3
0.050
Q1
0
Q2
0.39
Q4
8.91
Q3
90.7

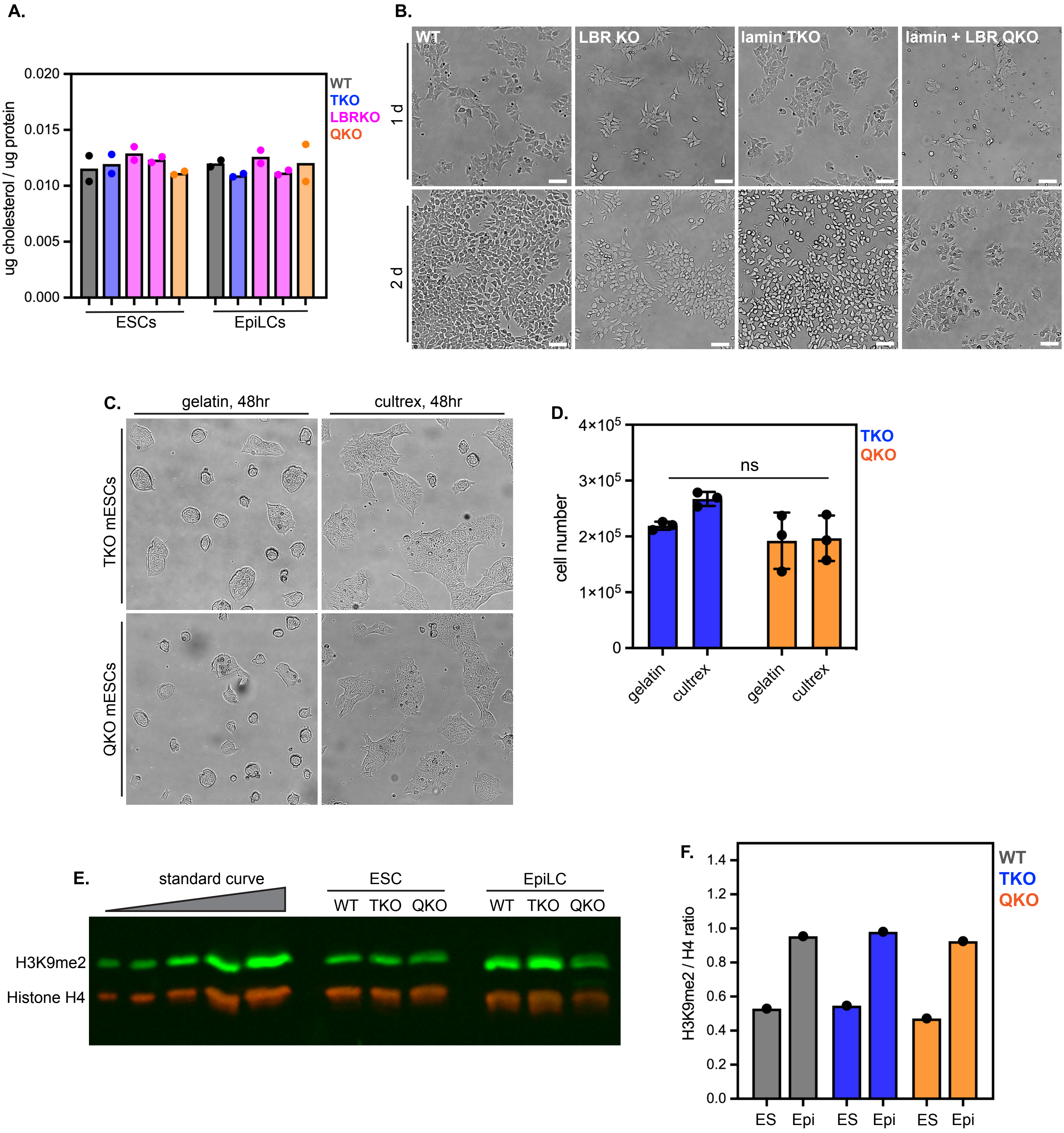
A.
ug cholesterol / ug protein
0.020
0.015
0.010
0.005
0.000
WT
TKO
LBRKO
QKO
ESCs
EpiLCs
B.
WT
LBR KO
lamin TKO
lamin + LBR QKO
1 d
2 d
C.
gelatin, 48hr
cultrex, 48hr
TKO mESCs
QKO mESCs
D.
cell number
4×10^5
3×10^5
2×10^5
1×10^5
0
TKO
QKO
ns
gelatin
cultrex
gelatin
cultrex
E.
standard curve
ESC
EpiLC
WT TKO QKO
WT TKO QKO
H3K9me2
Histone H4
F.
H3K9me2 / H4 ratio
1.4
1.2
1.0
0.8
0.6
0.4
0.2
0.0
WT
TKO
QKO
ES Epi ES Epi ES Epi

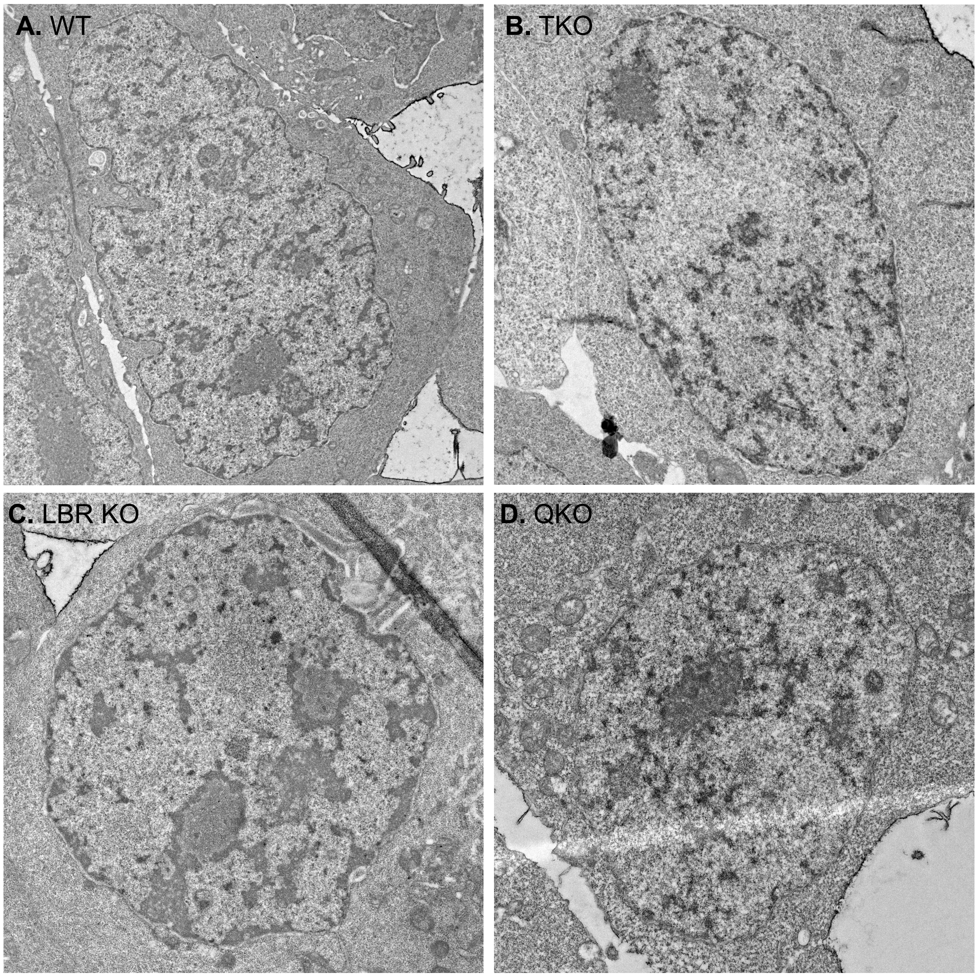
A. WT
B. TKO
C. LBR KO
D. QKO
