# Supplementary material for: The nuclear periphery confers repression on H3K9me2-marked genes and transposons to shape cell fate
Source: Nat Cell Biol. 2025 Jul 22;27(8):1311–26. doi: 10.1038/s41556-025-01703-z (PMC12339402; doi:10.1038/s41556-025-01703-z)
Supplement: Supplementary file 2 — Reporting Summary [file 41556_2025_1703_MOESM2_ESM.pdf]

nature portfolio

Corresponding author(s): Buchwalter, Abigail

Last updated by author(s): 2024-6-26

Reporting Summary

Nature Portfolio wishes to improve the reproducibility of the work that we publish. This form provides structure for consistency and transparency in reporting. For further information on Nature Portfolio policies, see our Editorial Policies and the Editorial Policy Checklist.

Statistics

For all statistical analyses, confirm that the following items are present in the figure legend, table legend, main text, or Methods section.

n/a	Confirmed	
☐	☒	The exact sample size (n) for each experimental group/condition, given as a discrete number and unit of measurement
☒	☐	A statement on whether measurements were taken from distinct samples or whether the same sample was measured repeatedly
☐	☒	The statistical test(s) used AND whether they are one- or two-sided<br>*Only common tests should be described solely by name; describe more complex techniques in the Methods section.*
☒	☐	A description of all covariates tested
☐	☒	A description of any assumptions or corrections, such as tests of normality and adjustment for multiple comparisons
☐	☒	A full description of the statistical parameters including central tendency (e.g. means) or other basic estimates (e.g. regression coefficient) AND variation (e.g. standard deviation) or associated estimates of uncertainty (e.g. confidence intervals)
☐	☒	For null hypothesis testing, the test statistic (e.g. F, t, r) with confidence intervals, effect sizes, degrees of freedom and P value noted<br>*Give P values as exact values whenever suitable.*
☒	☐	For Bayesian analysis, information on the choice of priors and Markov chain Monte Carlo settings
☒	☐	For hierarchical and complex designs, identification of the appropriate level for tests and full reporting of outcomes
☒	☐	Estimates of effect sizes (e.g. Cohen's d, Pearson's r), indicating how they were calculated

Our web collection on statistics for biologists contains articles on many of the points above.

Software and code

Policy information about availability of computer code

Data collection	The Nikon Elements software suite was used to acquire confocal microscopy data.
Data analysis	CellProfiler and Fiji open-source software were used for microscopy image analysis, and R and Graphpad Prism were used for further analysis and presentation of analyzed data. Standard R and Python packages and pipelines were used for analysis of transcriptomic and genomic data. Where previously published bioinformatic packages were used, citations are provided.

For manuscripts utilizing custom algorithms or software that are central to the research but not yet described in published literature, software must be made available to editors and reviewers. We strongly encourage code deposition in a community repository (e.g. GitHub). See the Nature Portfolio guidelines for submitting code & software for further information.

Data

Policy information about availability of data

All manuscripts must include a data availability statement. This statement should provide the following information, where applicable:

- Accession codes, unique identifiers, or web links for publicly available datasets
- A description of any restrictions on data availability
- For clinical datasets or third party data, please ensure that the statement adheres to our policy

Genomic and transcriptomic data generated during the course of this study were uploaded to the GEO database under the following accession numbers: mESC and EpiLC RNAseq data, GSE264599; LBR miR-E RNAseq data, GSE264602; H3K9me2 Cut & Run data, GSE264603; and LAP2b and LB1 Cut & R un data, GSE288122.

Research involving human participants, their data, or biological material

Policy information about studies with human participants or human data. See also policy information about sex, gender (identity/presentation), and sexual orientation and race, ethnicity and racism.

Reporting on sex and gender	n/a
Reporting on race, ethnicity, or other socially relevant groupings	n/a
Population characteristics	n/a
Recruitment	n/a
Ethics oversight	n/a

Note that full information on the approval of the study protocol must also be provided in the manuscript.

Field-specific reporting

Please select the one below that is the best fit for your research. If you are not sure, read the appropriate sections before making your selection.

☒ Life sciences ☐ Behavioural & social sciences ☐ Ecological, evolutionary & environmental sciences

For a reference copy of the document with all sections, see nature.com/documents/nr-reporting-summary-flat.pdf

Life sciences study design

All studies must disclose on these points even when the disclosure is negative.

Sample size	No power analyses were performed to predetermine sample size. For microscopy analyses, experiments were performed in at least 3 independent biological replicates with two technical replicates per experiment. For each biological replicate, numerous individual cells were analyzed and each cell is reported as an independent data point. For confocal microscopy experiments at least 10 cells were analyzed per biological replicate.<br>For differentiation experiments, at least 3 biological replicates and 2 technical replicates were performed.<br>For bulk RNAseq analyses, 4 replicates were performed. For Cut & Run analyses, 3 or 4 replicates were performed.<br>These sample sizes align with field standards.
Data exclusions	No data were excluded from any experiments included in this study.
Replication	All replicates of experiments produced consistent outcomes, and we had no issues with reproducing any findings shown in the manuscript in multiple independent experiments. A minimum of 3 replicates were performed.
Randomization	N/A. We did not use categorical measurements or scoring that would benefit from randomization.
Blinding	N/A. We did not use categorical measurements or subjective scoring that would benefit from blinding. Instead, we report quantitative measurements.

Reporting for specific materials, systems and methods

We require information from authors about some types of materials, experimental systems and methods used in many studies. Here, indicate whether each material, system or method listed is relevant to your study. If you are not sure if a list item applies to your research, read the appropriate section before selecting a response.

Materials & experimental systems

n/a	Involved in the study	
☐	☒	Antibodies
☐	☒	Eukaryotic cell lines
☒	☐	Palaeontology and archaeology
☒	☐	Animals and other organisms
☒	☐	Clinical data
☒	☐	Dual use research of concern
☒	☐	Plants

Methods

n/a	Involved in the study	
☐	☒	ChIP-seq
☒	☐	Flow cytometry
☒	☐	MRI-based neuroimaging

Antibodies

Antibodies used	H3K9me2 antibody (Abcam ab1220) was used for immunostaining and for Cut & Run.<br>H3K9me2 antibody (Active Motif 39041) was also used for immunostaining.<br>LAP2 antibody (Invitrogen PA5-52519) and LBR antibody (Abcam ab232731) were used for immunostaining.<br>SOX2 antibody (Rockland 610-1302) was used for Western blotting. Orf1p antibody (Abcam ab216324) was used for immunostaining.
Validation	The specificity of the H3K9me2 ab1220 antibody for H3K9me2 by ChIP-seq assays and of the H3K9me2 Active Motif antibody for immunostaining assays has been previously demonstrated (using competitor peptides) by Andrey Poleshko, Raj Jain, and colleagues (see Poleshko et al., Cell 2017 and eLife 2019). In addition, we verified that ab1220 and Active Motif H3K9me2 immunostaining signal disappears when cells are treated with the G9a/GLP inhibitor UNC0638.<br>The LAP2 antibody recognizes both the nucleoplasmic alpha and nuclear membrane-inserted beta isoforms, although the latter is much more readily detected by this antibody in mESCs. We have verified that LAP2 immunostaining at the nuclear periphery diminishes when LAP2b expression is inhibited by RNAi in human cells.<br>We verified the specificity of the LBR antibody by noting its lack of signal in LBR-null and LBR-RNAi conditions.

Eukaryotic cell lines

Policy information about cell lines and Sex and Gender in Research

Cell line source(s)	lamin TKO mESCs and wildtype littermate mESCs were the generous gift of Yixian Zheng and are described in their publication (Zheng et al., Mol Cell 2018). These mESCs are XY.
Authentication	wildtype and lamin TKO mESC cell lines were originally authenticated by the Zheng laboratory. We independently authenticated these lines by performing qPCR to validate lack of LMNA, LMNB1, and LMNB2 expression in lamin TKO mESCs.<br>LBR-null and lamin + LBR quadruple null mESCs were genotyped by PCR and Sanger sequencing, and lack of expression of detectable LBR was confirmed by immunostaining with multiple LBR antibodies.<br>Wild type, lamin TKO, and lamin + LBR QKO mESCs were karyotyped. These lines exhibited some karyotypic abnormalities that are frequently observed in stem cells which are reported in the Methods.
Mycoplasma contamination	wild type and lamin TKO mESCs were tested and confirmed to be mycoplasma negative.
Commonly misidentified lines<br>(See ICLAC register)	none used.

Plants

Seed stocks	n/a
Novel plant genotypes	n/a
Authentication	n/a

ChIP-seq

Data deposition

☒ Confirm that both raw and final processed data have been deposited in a public database such as GEO.

☒ Confirm that you have deposited or provided access to graph files (e.g. BED files) for the called peaks.

Data access links<br>*May remain private before publication.*	Here are three reviewer tokens for the three GEO entries:<br>https://www.ncbi.nlm.nih.gov/geo/query/acc.cgi?acc=GSE264599 token: ijuhaqkqlvadvij<br>https://www.ncbi.nlm.nih.gov/geo/query/acc.cgi?acc=GSE264602 token: ebofuiisxvqtvqx<br>https://www.ncbi.nlm.nih.gov/geo/query/acc.cgi?acc=GSE264603 token: mzgbkyworpkdjkn
Files in database submission	raw files:<br>LBRKO_epi_1_R1_001.fastq.gz<br>LBRKO_epi_1_R2_001.fastq.gz<br>LBRKO_epi_2_R1_001.fastq.gz<br>LBRKO_epi_2_R2_001.fastq.gz<br>LBRKO_epi_3_R1_001.fastq.gz<br>LBRKO_epi_3_R2_001.fastq.gz<br>LBRKO_naive_1_R1_001.fastq.gz

LBRKO_naive_1_R2_001.fastq.gz
LBRKO_naive_2_R1_001.fastq.gz
LBRKO_naive_2_R2_001.fastq.gz
LBRKO_naive_3_R1_001.fastq.gz
LBRKO_naive_3_R2_001.fastq.gz
QKO_epi_1_R1_001.fastq.gz
QKO_epi_1_R2_001.fastq.gz
QKO_epi_2_R1_001.fastq.gz
QKO_epi_2_R2_001.fastq.gz
QKO_epi_3_R1_001.fastq.gz
QKO_epi_3_R2_001.fastq.gz
QKO_naive_1_R1_001.fastq.gz
QKO_naive_1_R2_001.fastq.gz
QKO_naive_2_R1_001.fastq.gz
QKO_naive_2_R2_001.fastq.gz
QKO_naive_3_R1_001.fastq.gz
QKO_naive_3_R2_001.fastq.gz
TKO_epi_1_R1_001.fastq.gz
TKO_epi_1_R2_001.fastq.gz
TKO_epi_2_R1_001.fastq.gz
TKO_epi_2_R2_001.fastq.gz
TKO_epi_3_R1_001.fastq.gz
TKO_epi_3_R2_001.fastq.gz
TKO_naive_1_R1_001.fastq.gz
TKO_naive_1_R2_001.fastq.gz
TKO_naive_2_R1_001.fastq.gz
TKO_naive_2_R2_001.fastq.gz
TKO_naive_3_R1_001.fastq.gz
TKO_naive_3_R2_001.fastq.gz
WT_epi_1_R1_001.fastq.gz
WT_epi_1_R2_001.fastq.gz
WT_epi_2_R1_001.fastq.gz
WT_epi_2_R2_001.fastq.gz
WT_epi_3_R1_001.fastq.gz
WT_epi_3_R2_001.fastq.gz
WT_naive_1_R1_001.fastq.gz
WT_naive_1_R2_001.fastq.gz
WT_naive_2_R1_001.fastq.gz
WT_naive_2_R2_001.fastq.gz
WT_naive_3_R1_001.fastq.gz
WT_naive_3_R2_001.fastq.gz

processed files:
LBRKO_epi_1_RPKM_10kb.bw
LBRKO_epi_1_RPKM_1kb.bw
LBRKO_epi_2_RPKM_10kb.bw
LBRKO_epi_2_RPKM_1kb.bw
LBRKO_epi_3_RPKM_10kb.bw
LBRKO_epi_3_RPKM_1kb.bw
LBRKO_naive_1_RPKM_10kb.bw
LBRKO_naive_1_RPKM_1kb.bw
LBRKO_naive_2_RPKM_10kb.bw
LBRKO_naive_2_RPKM_1kb.bw
LBRKO_naive_3_RPKM_10kb.bw
LBRKO_naive_3_RPKM_1kb.bw
QKO_epi_1_RPKM_10kb.bw
QKO_epi_1_RPKM_1kb.bw
QKO_epi_2_RPKM_10kb.bw
QKO_epi_2_RPKM_1kb.bw
QKO_epi_3_RPKM_10kb.bw
QKO_epi_3_RPKM_1kb.bw
QKO_naive_1_RPKM_10kb.bw
QKO_naive_1_RPKM_1kb.bw
QKO_naive_2_RPKM_10kb.bw
QKO_naive_2_RPKM_1kb.bw
QKO_naive_3_RPKM_10kb.bw
QKO_naive_3_RPKM_1kb.bw
TKO_epi_1_RPKM_10kb.bw
TKO_epi_1_RPKM_1kb.bw
TKO_epi_2_RPKM_10kb.bw
TKO_epi_2_RPKM_1kb.bw
TKO_epi_3_RPKM_10kb.bw
TKO_epi_3_RPKM_1kb.bw
TKO_naive_1_RPKM_10kb.bw
TKO_naive_1_RPKM_1kb.bw
TKO_naive_2_RPKM_10kb.bw

TKO_naive_2_RPKM_1kb.bw
TKO_naive_3_RPKM_10kb.bw
TKO_naive_3_RPKM_1kb.bw
WT_epi_1_RPKM_10kb.bw
WT_epi_1_RPKM_1kb.bw
WT_epi_2_RPKM_10kb.bw
WT_epi_2_RPKM_1kb.bw
WT_epi_3_RPKM_10kb.bw
WT_epi_3_RPKM_1kb.bw
WT_naive_1_RPKM_10kb.bw
WT_naive_1_RPKM_1kb.bw
WT_naive_2_RPKM_10kb.bw
WT_naive_2_RPKM_1kb.bw
WT_naive_3_RPKM_10kb.bw
WT_naive_3_RPKM_1kb.bw
cutandrun_counts_TE_unique.txt
cutandrun_counts_genes.txt

Genome browser session (e.g. UCSC)

n/a

Methodology

Replicates

Three replicates of each genotype were performed for Cut & Run analyses. All replicates clustered together in unsupervised hierarchical clustering, and these data are reported in Supplementary Figures 4 and 9.

Sequencing depth

For Cut & Run, approximately 16 million reads were sequenced per library (paired-end sequencing, 35bp read length)
For RNAseq, approximately 30 million reads were sequenced per library (paired-end sequencing, 100 bp read length).

Antibodies

H3K9me2 antibody ab1220 from Abcam and rabbit anti-mouse secondary antibody (Abcam ab6709) wer used for Cut & Run experiments.

Peak calling parameters

Cut & Run reads were mapped using Bowtie2 and filtered with SAMtools. H3K9me2 domains were called using a Hidden Markov Model approach via the Pomegranate Python package.

Data quality

Cut & Run data has a significantly higher dynamic range and lower background than ChIP-seq data, and fold-enrichment cutoffs or FDR cutoffs are less frequently needed for this type of data. (see work from Henikoff & colleagues in eLife 2017 and Nat Comms 2019.)

Software

Cut & Run data were mapped and analyzed using published bioinformatic pipelines and packages in R and Python; previously published packages used are cited in the Methods section.
