# Supplementary material for: The nuclear periphery confers repression on H3K9me2-marked genes and transposons to shape cell fate
Source: Nat Cell Biol. 2025 Jul 22;27(8):1311–26. doi: 10.1038/s41556-025-01703-z (PMC12339402; doi:10.1038/s41556-025-01703-z)
Supplement: Supplementary file 6 — Unprocessed western blots and gels. [file 41556_2025_1703_MOESM6_ESM.pdf]

Supplementary Figure 1

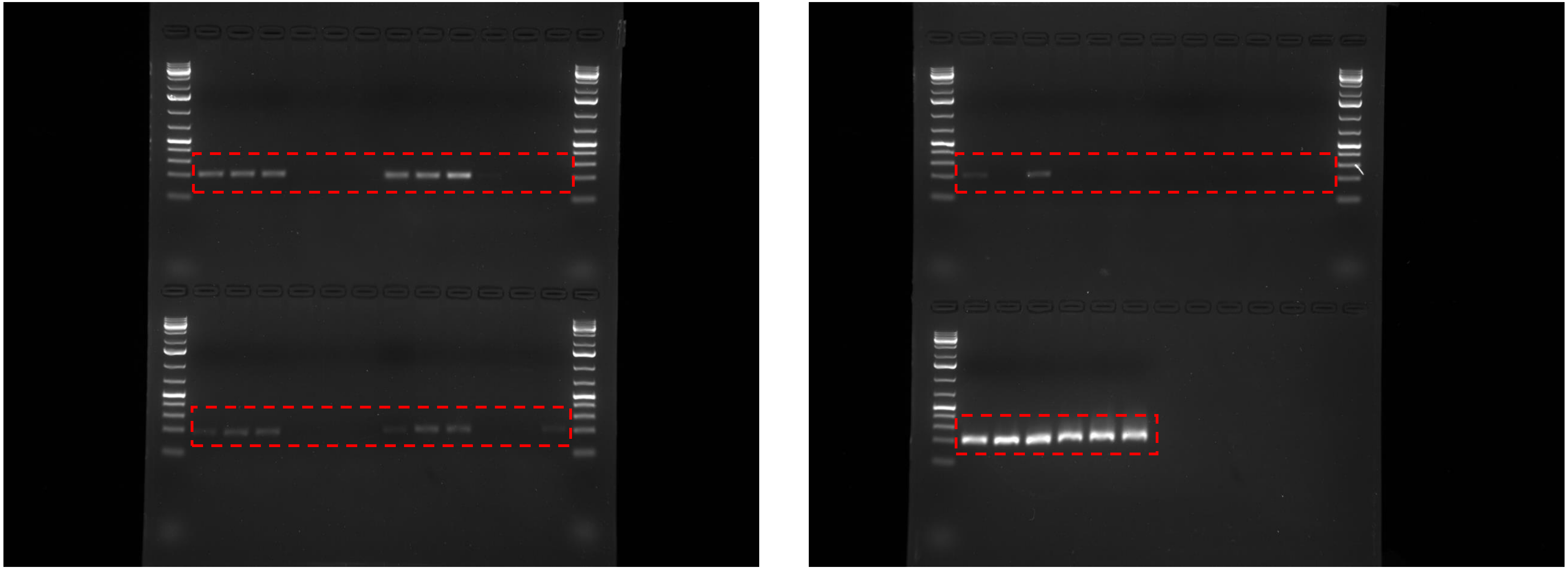

Supplementary Figure 2E and 2F

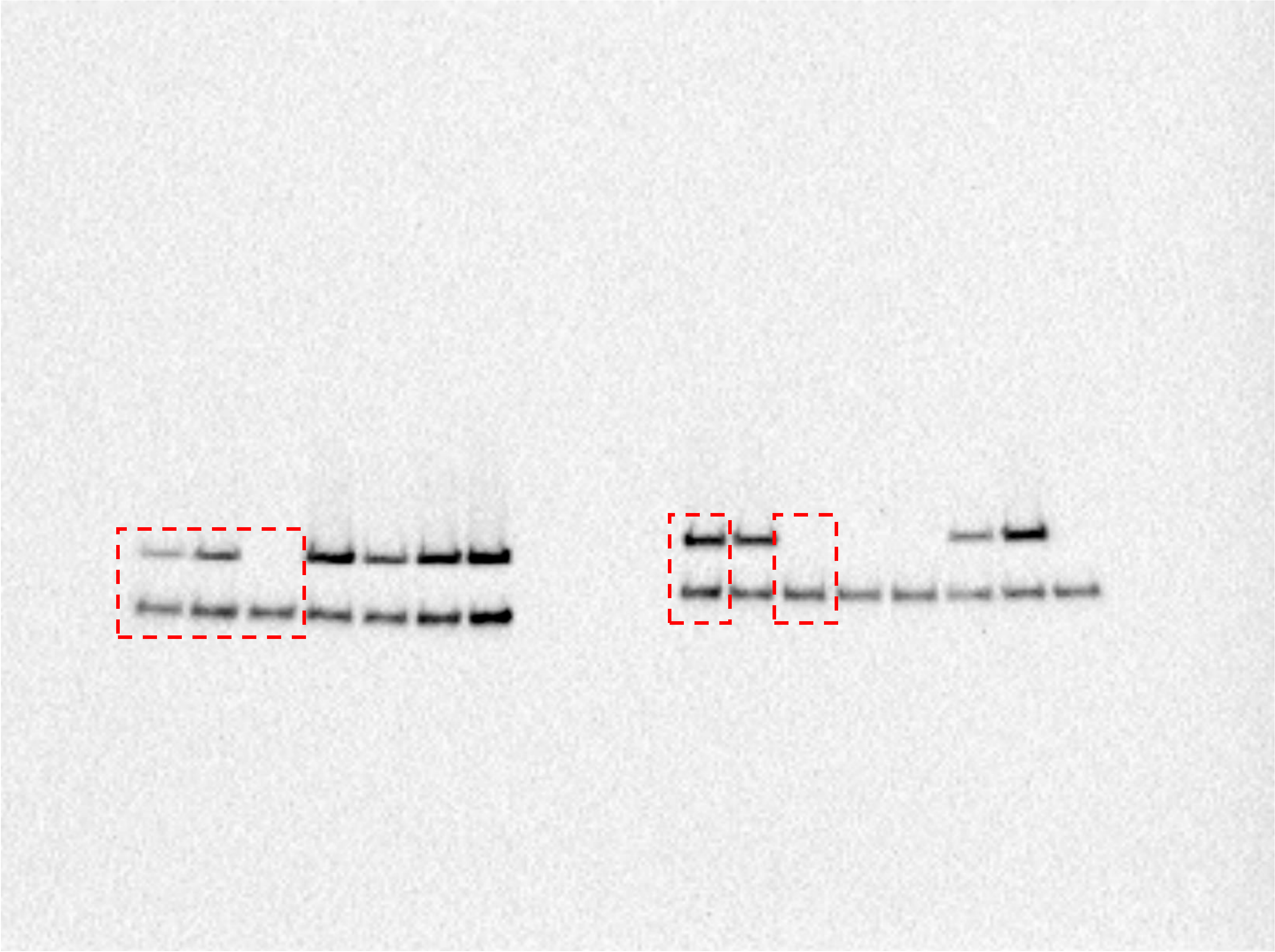

Extended data figure 3F

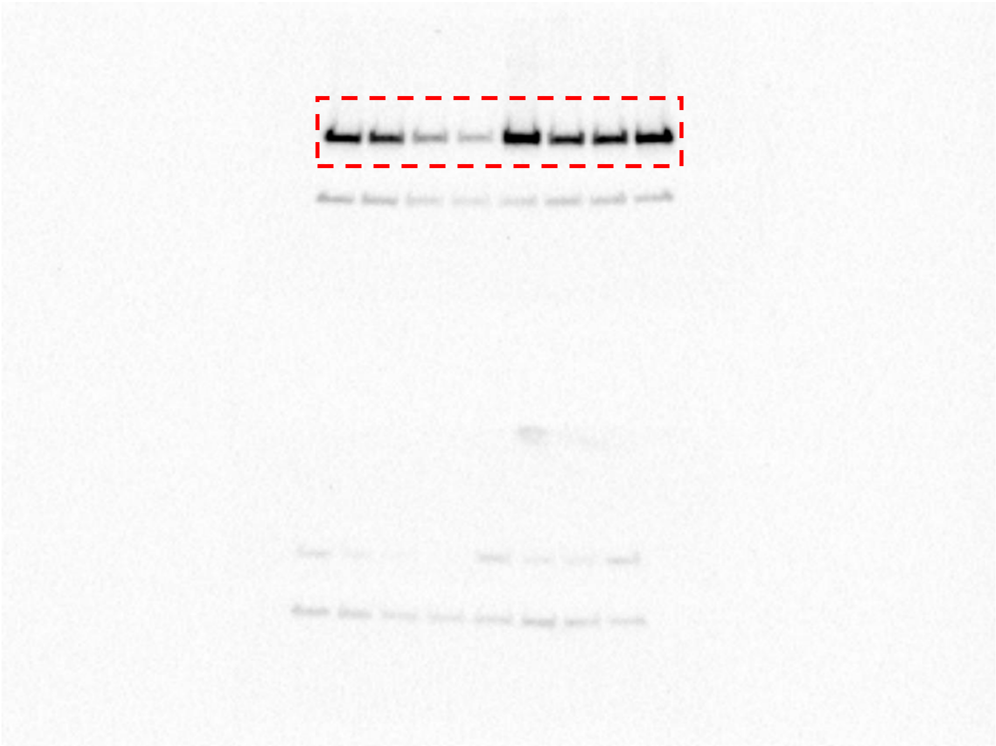

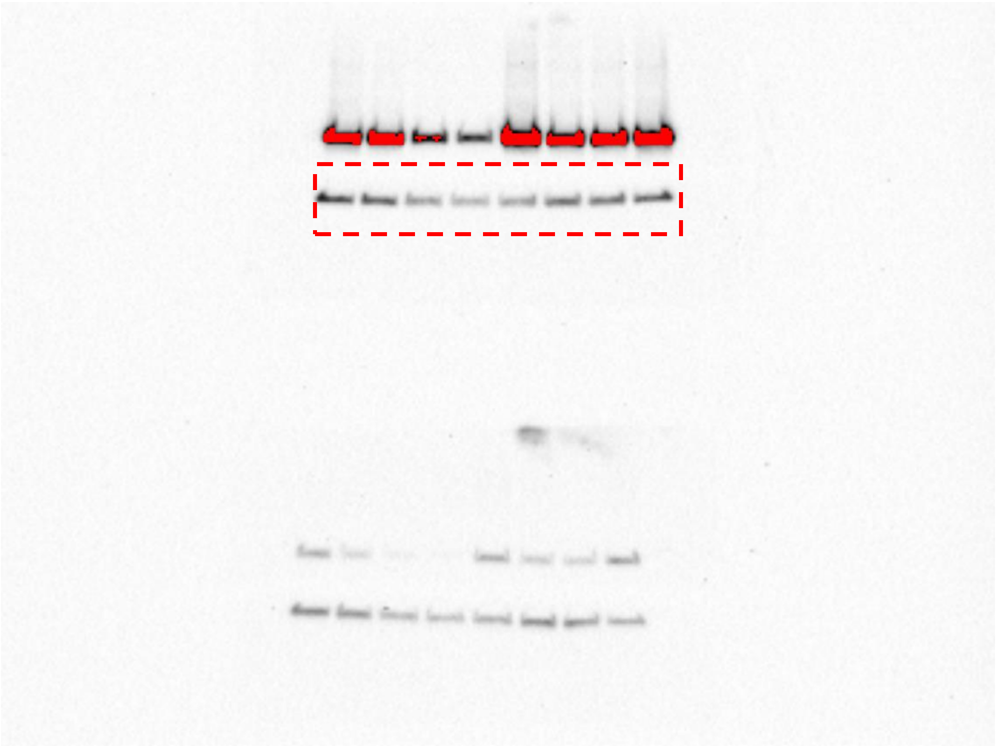

Supplementary Figure 5D and 5E

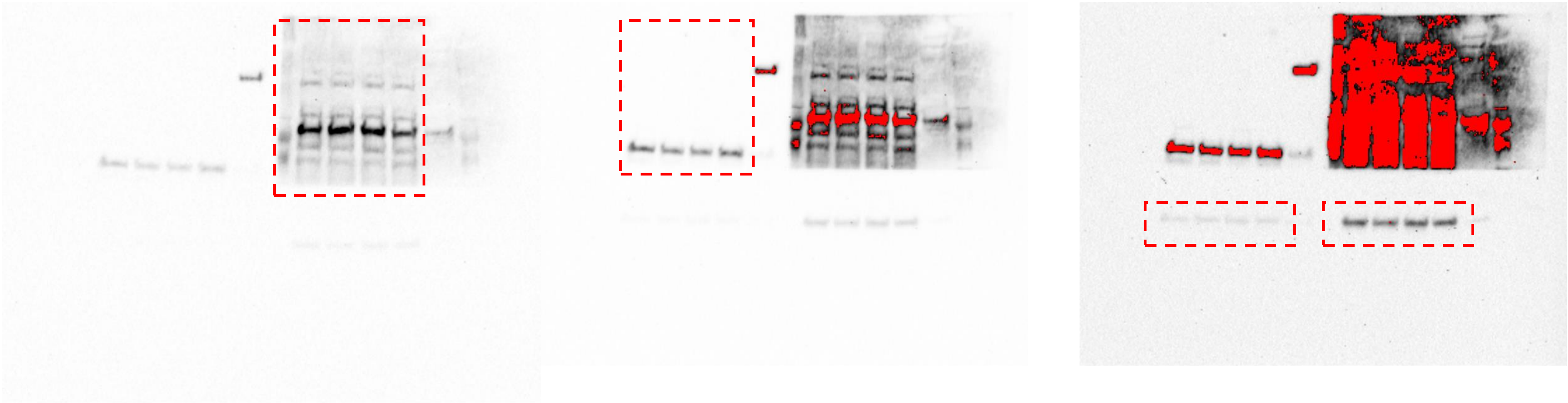

Supplementary Data Fig 7E

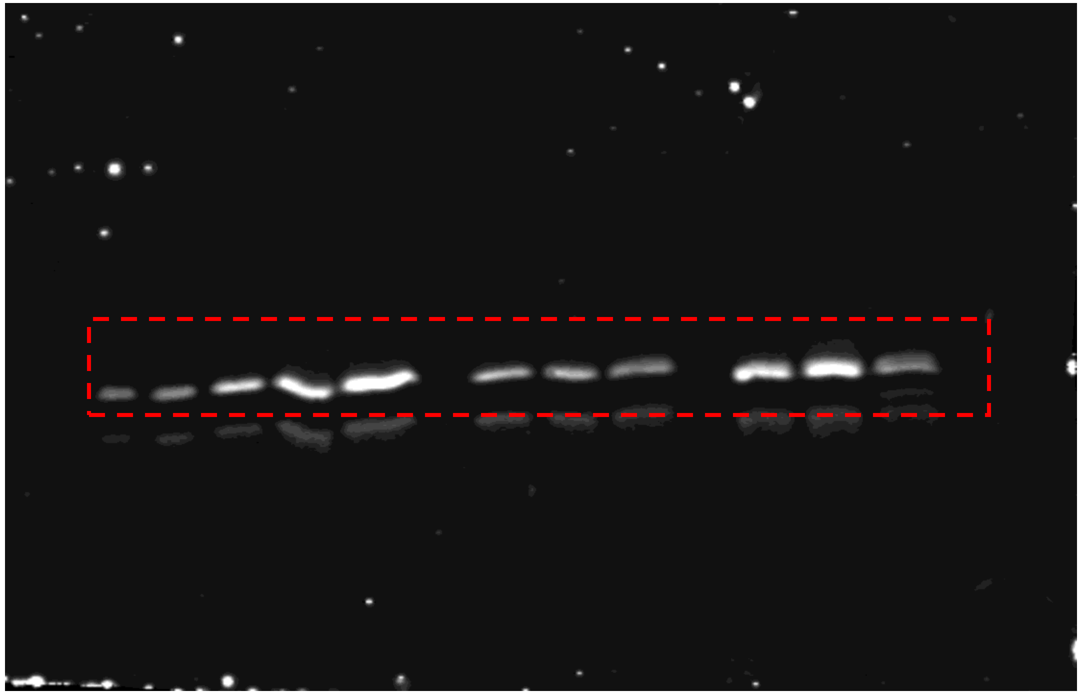

H3K9me2 channel

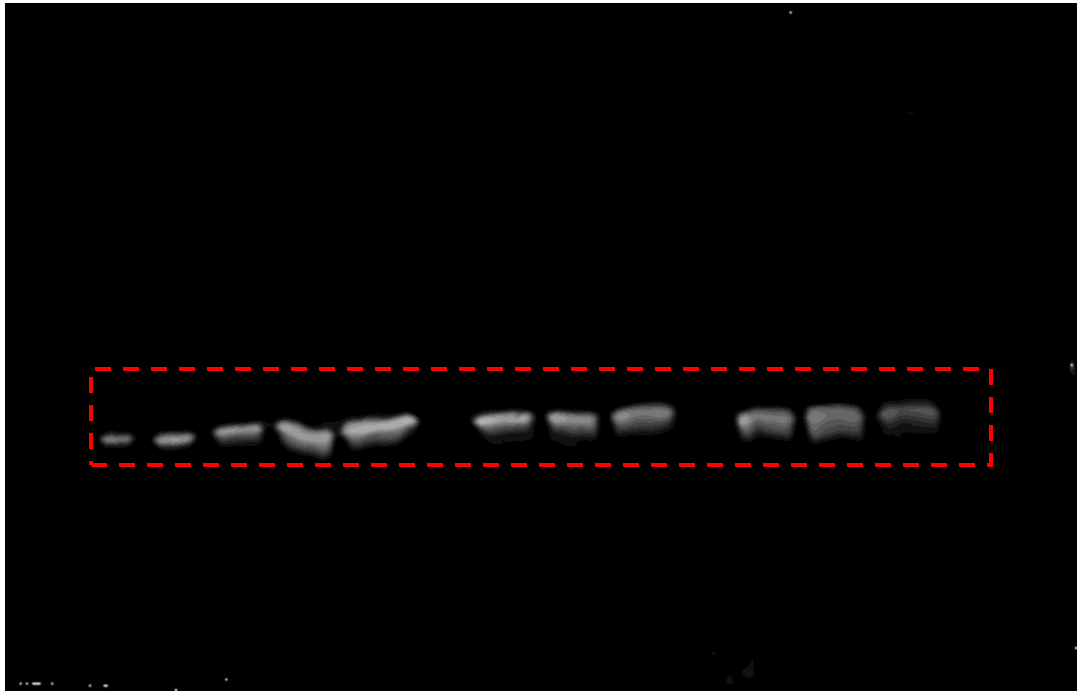

H4 channel
